# Small Family with Key Contacts: Par14 and Par17 Parvulin Proteins, Relatives of Pin1, Now Emerge in Biomedical Research

**DOI:** 10.4137/pmc.s496

**Published:** 2008-03-07

**Authors:** Jonathan W Mueller, Peter Bayer

**Affiliations:** 1 Institute for Structural and Medicinal Biochemistry, Centre for Medical Biotechnology—ZMB, University of Duisburg-Essen, 45117 Essen, Germany; 2 Molecular Structure, National Institute for Medical Research (MRC), The Ridgeway, NW7 1AA, London, U.K

## Abstract

The parvulin-type peptidyl-prolyl *cis/trans* isomerase Pin1 is subject of intense biochemical and clinical research as it seems to be involved in the pathogenesis of certain cancers and protein folding illnesses like Alzheimer’s and Parkinson’s disease. In addition to Pin1, the human genome only contains a single other parvulin locus encoding two protein species—Par14 and Par17. Much less is known about these enzymes although their sequences are highly conserved in all metazoans. Parvulin has been proposed to function as Pin1 complementing enzyme in cell cycle regulation and in chromatin remodelling. Pharmaceutical modulation of Par14 might therefore have benefits for certain types of cancer. Moreover, the Par17 protein that has been shown to be confined to anthropoid primate species only might provide a deeper understanding for human-specific brain development. This review aims at stimulating further research on Par14 and Par17 that are overlooked drug targets in the shadow of an overwhelming plethora of Pin1 literature by summarising all current knowledge on these parvulin proteins.

## Introduction

Protein mediated biological processes are strictly regulated in a spatial and temporal manner. This regulation is strongly affected by peptidyl prolyl *cis/trans* isomerases (PPIases) both at the stage of protein folding as well as within the native state.^1^ There are PPIases that bind to immunosuppressant drugs and hence are referred to as immunophilins.^2^,^3^ These are the cyclophilins which bind to cyclosporin A and the FKBPs (FK506 binding proteins) which are inhibited by FK506 and rapamycin. Parvulins are a third class of PPIases conserved from bacteria to man. A prominent parvulin is the mitotic regulator Pin1^4^ involved in cell cycle regulation and protein folding disorders such as Alzheimer’s or Parkinson’s disease. The vast amount of literature about this phosphorylation specific PPIase has been reviewed numerous times, here only giving some of the most recent references.^5^–^12^

The human genome contains one more parvulin gene besides Pin1—a locus on Chromosome Xq13 encoding two protein species, Par14 and Par17.^13^ The core sequence of these parvulins is highly conserved in all multi-cellular organisms examined so far, but absent from yeasts. Within the human genome two intron-less parvulin pseudogenes on chromosome 1 and 15 can be found;^13^ but since they are truncated at the 5′ end and they contain several point mutations, they are not expressed. The occurrence of Par17 has furthermore been shown to be confined to the species of great apes and humans only^14^ and thus might provide a deeper understanding of human-specific brain development. Parvulin proteins might therefore turn out to be valuable drug targets besides the well known mitotic regulator protein Pin1.

## Identification of Parvulin Proteins

The first member of the parvulin family of PPIases was the *E. coli* enzyme Par10.^15^,^16^ It is a cytosolic protein solely consisting of the PPIase domain with no additional N- or C-terminal extensions. Its name was derived from the Latin word *parvulus* (meaning *tiny*) due to its low molecular weight of about 10 kDa as compared to the larger FKBPs and cyclophilins. The substrate specificity of Par10 resembles that of FKBP isomerases with a preference for bulky, hydrophobic side chains preceding the proline residue;^17^ the cellular function of this protein is still elusive.

Human Pin1 was then identified by two-hybrid screening as a protein interacting with NIMA kinase;^4^ in addition, Pin1 suppresses the growth inhibiting effect of this kinase. Using tetrad analysis as well as plasmid shuffling experiments the same study showed that human Pin1 can substitute for yeast Ess1 (see below) both in haploid and diploid cells.^4^ Depletion of Pin1 from yeast or HeLa cells induced mitotic arrest^4^ and Pin1 inhibition caused apoptosis in a ras-transformed tumour cell line^18^ whereas HeLa cells over-expressing Pin1 arrest in G2 phase of the cell cycle. Pin1^−/−^ mice are viable but show cell cycle defects resembling cyclin D1 knockout^19^,^20^ and defects in spermatogenesis.^21^,^22^ Pin1 has a strong preference for phosphorylated serine or threonine side chains preceding proline.^23^

The only yeast parvulin, Ess1, was cloned way before the identification of Pin1 as a protein essential for growth,^24^ however its enzymatic activity as a PPIase was only described later.^25^,^26^ One of its major cellular substrates seems to be the multi-phosphorylated C-terminal domain of RNA polymerase II^27^,^28^ as this isomerase shares the preference for phosphorylated Ser/Thr-Pro motifs. Intriguingly, the essential function of Ess1 is already fulfilled at 0.5 per cent of wild type protein levels.^29^ The homologous protein in *Candida albicans* is essential for growth and morphogenetic switching,^30^ while the corresponding one in *Cryptococcus neoformans* is not required for normal growth and only needed for virulence.^31^ Pin1/Ess1 related parvulin sequences are now available from a variety of different eukaryotic organisms.^32^

### Human parvulins different from Pin1

Another human parvulin was cloned as a homologue of human Pin1 and *E. coli* parvulin Par10. It consists of a 1.0 kilo-base cDNA encoding a 156 amino acids protein named Par14^33^ or Eukaryotic Homolog of Parvulins (EHPV)^34^ and is assumed to be involved in cell cycle progression or chromatin remodelling.^35^,^36^ For unknown reasons, the name “Pin4” was assigned to this protein in public databases meaning “protein interacting with NIMA kinase 4”; however, such interaction has never been shown for Par14. The protein contains a C-terminal PPIase domain that has 34% and 39% sequence identity with the PPIase domains of *E. coli* Par10 and human Pin1, respectively. In addition, Par14 has an N-terminal flexible domain rich in lysine, serine and glycine residues.^37^,^38^

Northern blot analysis of human Par14 expression using the very same multiple tissue membranes in two studies^33^,^34^ showed an enhanced expression in heart and skeletal muscles but weak signals in brain and lung tissues. Par14 expression in placenta, liver, kidney and pancreas was at moderate levels. In addition, an mRNA dot-blot showed Par14 expression in a variety of tissues, but notably at relatively lower levels in neuronal tissues.^34^

Parvulin 14 was proposed to partially compensate for Pin1 loss in mammalian cells as its mRNA and protein levels were up-regulated in Pin1^−/−^ mouse endothelial fibroblasts (MEFs).^39^ The very same study purported that siRNA depletion of Par14 inhibited growth of Pin1^−/−^ MEFs much stronger than Pin1^−/−^ MEFs re-expressing Pin1.^39^ Furthermore, Par14 has been proposed as a Pin1 complementing enzyme in cell cycle regulation^39^ and chromatin remodelling.^35^ However, compensation for Pin1 loss is not complete as Par14 failed to rescue the deletion of the yeast parvulin Ess1.^40^ Moreover, its comparably low activity was not specific for phosphorylated peptide motifs.^33^ This suggests divergent, but partially overlapping cellular functions for the two human parvulins. Complementation or cross talk between different PPIases has already been studied in yeast. There, the cyclophilin CypA is able to interact with and complement for the yeast parvulin Ess1.^27^,^41^,^42^

**Figure 1:**
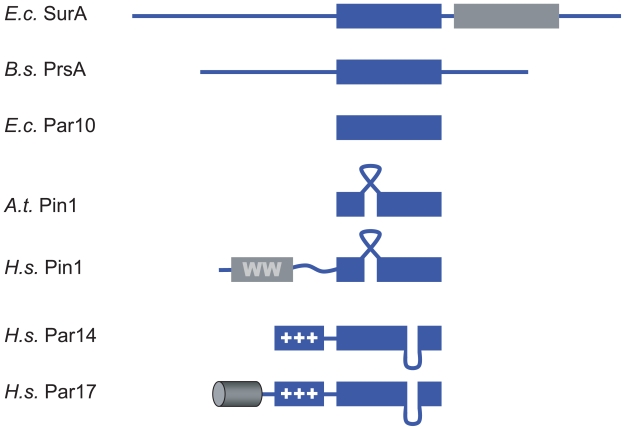
Schematic overview on different parvulin proteins Bacterial, plant and human parvulin representatives are depicted with their domain structure. *E. coli* SurA contains two PPIase domains (blue and gray) and large N- and C-terminal extensions. *E. coli* Par10 does not contain any extensions or additional loops—as one of the scarce parvulin sequences from Archaea, PinA from *Cenarchaeum symbiosum* (SwissProt: O74049). There are phospho-specific parvulins (indicated with the ribbon-like loop) both with and without WW domain. The WW domain is connected to the catalytic domain by a flexible linker in human Pin1.^66^ Par14-like parvulins contain an N-terminal basic domain (+++) and a five amino acid insertion (loop) between the C-terminal helix and beta-strand of the PPIase domain. An alpha-helical extension (barrel) to this sequence is hominid-specific.

Besides Par14, a second protein species is encoded by the parvulin gene on chromosome Xq13 with an elongated N-terminus.^13^ The extended mRNA of Par17 is only transcribed in low copy numbers compared to Par14 with about 0.5% of total parvulin mRNA in skeletal muscle and liver tissues and up to 1.5% in brain and epithelial tissues.^13^ The Par17 protein is targeted to the mitochondrial matrix with the N-terminal domain acting as mitochondrial targeting peptide.^14^ As this mitochondrial targeting signal is only encoded within the genomes of great apes and humans, this might be the most recently evolved cellular targeting peptide known to date.^14^ The domain structure of different parvulin proteins is shown in Figure 1.

### The emergence of two parvulin genes within Eukarya

**Figure 2:**
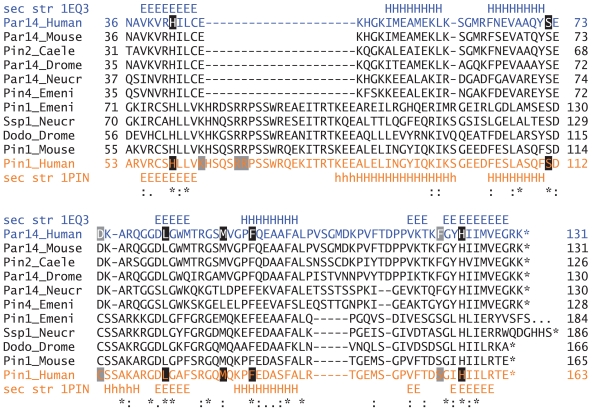
Alignment of parvulin and Pin1 sequences from different multicellular organisms Pin1 and Par14 structures 1PIN and 1EQ3, respectively, were aligned by DALILite.^67^ Following sequences were obtained from SwissProt and added to this alignment: Par14_ Human, Q9Y237; Par14_Mouse, Q9CWW6; Pin2_Caele, Q9NAF9 (Y48C3A.16); Par14_Drome, Q9VBU4 (CG11858); Par14_Neucr, Q7RYY4; Pin4_Emeni, Q5B5W1 (Aspergillus nidulans or Emericella nidulans); Pin1_Emeni, Q5AZY5; Ssp1_Neucr, Q7RVY7; Dodo_Drome, P54353; Pin1_Mouse, Q9QUR7; Pin1_Human, Q13526. The start of the PPIase domain within these sequences is given. All but Pin1_Emeni end at the position indicated by an asterisk (*). The Pin1_Emeni sequence contains 26 additional residues. 1EQ3, 1PIN1: PDB entries where the secondary structure information was taken from. Residues believed to be important for PPIase activity are highlighted in black when conserved between Par14 and Pin1, otherwise in gray.

Pin1 and Par14 sequences are found in all multicellular organisms from *N. crassa* and *C. elegans* to man, whereas yeasts contain only one parvulin homolog called Ess1. Compared with the cyclophilin and FKBP repertoires, the parvulin repertoires of eukaryotic organisms are relatively small with just two parvulins in most metazoans.^32^ One might therefore ask when this second parvulin was acquired. A detailed analysis of the PPIase repertoires of a variety of fungal genomes confirms the existence of a single parvulin gene within the phylogenetic clade Saccharomycotina that includes for example *Saccharomyces cerevisiae* or *Candida albicans*; only in the clade of Pezizomycotina including for instance *Aspergillus nidulans* or *Neurospora crassa* two parvulin genes are found.^43^ Since then, parvulin coding sequences have been highly conserved as seen on the alignment in Figure 2 with the Par14 sequences from human and *A. nidulans* sharing 55% and the Pin1 sequences 57% identity on the amino acid level. In contrast, the parvulin-type PPIases only share 24% and 32% identity within *A. nidulans* or humans, respectively. Hence, studying parvulin proteins in filamentous fungi could yield valuable insights into their cellular functions.

## Comparison of Structure and Mechanism of Parvulin Proteins

The primary structure of the Par14 and Pin1 PPIase domains show striking differences between the two when aligned with the ClustalW program (not shown). The large phospho-binding loop of Pin1 is missing in Par14; this protein instead has an insertion of five amino acids near the C-terminal end of the PPIase domain. Notably, a coupled exchange of residues can be observed within the N-terminal β-sheet: Cys57 and Val62 in all Pin1 sequences are replaced by Val40 and Cys45 in all Par14-type proteins. More elusive than the ClustalW alignment was a structure-based comparison of the PPIase domains including phylogenetic information (Fig. 2). From this figure it becomes clear that conserved residues between Par14 and Pin1 mainly locate to secondary structure elements. In addition, the Par14 specific insertion of five amino acids C-terminal to the last helix is contained in all Par14 sequences but without obvious conservation of sequence.

The NMR solution structure of Par14 was solved independently by two groups (PDB accession numbers 1EQ3^37^ and 1FJD^38^). The global fold of the PPIase domain consists of a twisted four-stranded β-sheet wrapping around the C-terminal helix; the other helices stacking on the other side of the central β-sheet. Structural similarities to FKBP12 and Pin1 were identified in Par14 as its secondary structure elements show the same topological arrangement as in Pin1 crystal structure 1PIN.^37^,^44^ An overlay of Pin1 and Par14 1EQ3 structures as well as a comparison between the two Par14 structures is depicted in Figure 2A and B.

When compared to the structure of the eponymous parvulin Par10 from *E. coli* (1JNT^45^), Par14 shares nearly all secondary features with this bacterial parvulin. Worth mentioning, Par10 contains a Gly76-Pro77 dipeptide in *cis* conformation^45^ structurally corresponding to Asp113-Pro114-Pro115 in the Par14 structures where such strong constrains towards the *cis* conformation has not been observed. Hitherto, more parvulin structures are available from *Bacillus subtilis* PrsA (1ZK6^46^), *Arabidopsis thaliana* Pin1 (1J6Y^47^), *E. coli* SurA (1M5Y^48^) and *Candida albicans* Ess1 (1YW5^49^). Although the parvulin structures from bacteria and fungi may be used for the development of antibacterial and anti-fungal drugs, none of the structures mentioned has an insertion corresponding to Par14 Val103 to Asp107. Hence, this insertion seems to be confined to parvulins from multicellular eukaryotes only.

### The Par14 specific loop region as a putative protein docking site

Par14-type PPIase sequences from metazoans contain a specific extension between the third β-strand and the C-terminal α-helix, but a functional role for this part of the protein has not been discussed so far. This region is at least 10 Å away from the putative active site of the protein.^37^ Hence, a role in specific interaction with other biopolymers is more likely rather than a direct involvement in substrate binding. The Val103-Asp107 insertion constitutes a flexible loop surrounded by hydrophobic side chains of Met55, Leu58, Met106, Pro109, Val110, Phe111, Pro114 and Met126^37^ suggesting this part of Par14 to be a potential docking site for other proteins.

The two Par14 solution structures differed in the very same region between amino acids Pro102 and Pro114 (see comparison of 1EQ3 and 1FJD in Figure 2B). As mentioned above, the structure of the *E. coli* homologue^45^ contains a Gly-Pro dipeptide exclusively in *cis* conformation at the position corresponding to Asp113-Pro114-Pro-115 (Fig. 2C). Therefore, the observed structural heterogeneity seen at this site of Par14 might represent native-state proline isomerisation. Whether a crystallographic analysis of Par14 reveals an exact and non-ambiguous geometry of this loop region remains to be determined in the future.

### Dividing parvulin proteins into subfamilies

From an enzymatic point of view, one can divide parvulin PPIases into at least two subfamilies.^17^ Clearly, one group prefers phosphorylated substrates as has been demonstrated for human Pin1, yeast Ess1 and some plant parvulins. Concomitant to the preference of phosphorylated substrates, the Pin1-like parvulins comprise the above mentioned loop between the N-terminal β-strand and α-helix of the PPIase domain (see Fig. 1) that is involved in phospho-peptide recognition. All these enzymes display exceptionally high catalytic activities (k_cat_/K_M_) towards Ser/Thr-Pro substrates modified by phosphorylation.^17^ The presence or absence of a WW domain N-terminal to the PPIase is not sufficient to assign a parvulin to this class of enzymes as deletion of Pin1’s WW domain has no effect on the *in vitro* activity of the enzyme.^50^ A more meaningful criterion for assigning parvulins to the Pin1-like sub-group is the ability to complement the otherwise lethal Ess1 loss in *S. cerevisiae.*^40^,^51^

To group the remaining parvulins is a rather difficult attempt. They have been described as a group of proteins with varying substrate specificity and with poorly characterised cellular function^46^ with human Par14 and the bacterial parvulins belonging to this subfamily. The observed k_cat_/K_M_ values varied within a range from 10^3^ to 10^7^ M^−1^ s^−1^ for several such parvulins.^17^ To pool all these parvulins together in one other subfamily just on the basis of lack of knowledge neglects the very different catalytic activities of certain parvulins. Human Par14 and *E. coli* Par10, for instance, are two enzymes sharing a rather unspecific substrate recognition pattern.^40^ Apparently, these two enzymes differ a lot from each other: *E. coli* Par10 displayed with 1.35 × 10^7^ M^−1^ s^−1^ towards the peptidic substrate Suc-ALPF-pNA an enzymatic activity comparable to human Pin1 whereas Par14’s reported activity of 3.9 × 10^3^ M^−1^ s^−1^ towards Suc-ARPF-pNA was at a lower limit of detection.^33^ Nevertheless, *E. coli* Par10 does catalyse the proline-limited folding of a variant of ribonuclease T1 at least 100-fold slower than the isomerization of the above mentioned peptide.^52^ The real substrate for Par14 might have escaped identification so far.

### Catalytic residues in parvulin proteins

Residues essential for catalysis have been proposed for Pin1-like parvulins based on a complex structure of Pin1 with an Ala-Pro dipeptide and a sulphate bound to the active site^44^ in line with site-directed mutagenesis experiments.^26^,^44^ H59, C113, S154 and H157 have been observed close to the peptide bond of the ligand. Some of the residues of the putative substrate binding pocket are not contained in the other group of parvulins as seen in Figure 1 with Pin1 S154 being exchanged by F120 in the Par14 sequence.

The most notable difference is Cys113 of Pin1 being replaced by Asp74 in Par14.^33^ This Cys113’s involvement in the catalysis of Pin1 was postulated to act as a nucleophile. Pin1 Cys133Ala and *E. coli* Par10 Cys41Ala mutants displayed more than 100-fold reduced catalytic activities compared to the wild-type.^44^ Because difficulties have been reported in detecting very low isomerase activities,^34^ one could ask whether parvulins with an aspartic acid at the corresponding position are active PPIases at all. Their isomerase activity towards peptidic substrates is rather small, but definitively has been observed with Par14,^33^ SurA^53^ and PrsA.^54^ In addition, mutating the Asp residue in PrsA to Ala (D154A) resulted in a protein with 50% remaining activity towards the peptide Suc-AKPF-pNA relative to wild-type PrsA.^46^,^54^

The role of several residues has recently been challenged formerly believed to be crucial for Pin1 function. In a mutagenesis screen, Behrsin and colleagues isolated a variety of Pin1 mutants which were still able to functionally replace yeast Ess1.^55^ Replacement of C113 by serine markedly reduced Pin1 function^55^ as shown before.^44^ Exchanging this very cysteine with an aspartic acid however resulted in no detectable loss in Pin1 function both in yeasts and in the purified recombinant protein.^55^ In spite of the surprising properties of the C113D mutant, a C113N mutation resulted in an inactive enzyme. As a result, cysteine and aspartic acid at this position are both compatible with PPIase activity and the role for C113 as a nucleophile within the catalytic process^17^ is worth to be reconsidered. More structures of FKBP and parvulin proteins in complex with putative substrates might yield further insights into the catalytic mechanism of these enzymes.

## What is the Function of Par14?

Despite eight years since its discovery, the function of Par14 still remains obscure. There are no known naturally occurring mutations associated with the human parvulin locus on chromosome Xq13. We are not aware of any knockout study of this protein in mice. Moreover, RNA*i* against the respective Par14 homologue have not caused an observable phenotype in two large-scale *C. elegans* RNA*i* screens. One searched for embryonic lethality, abnormal morphology or maternal sterility,^56^ the other for the phenotypes abnormal postembryonic development or lethality.^56^,^57^ Taken the very same degree of conservation between Par14 and Pin1 proteins within multi-cellular organisms and the partial compensation for Pin1 loss, Par14 is rather unlikely a spare coding sequence but its function is yet to be revealed in the future.

In evolutionary terms it could have been necessary to have highly active unspecific parvulins in primordial cells. Nowadays, the prokaryotic parvulins might resemble these common ancestors most closely. More specific parvulin PPIases could have emerged within the evolution of Eukarya. Pin1-type parvulins fulfil the need to isomerise special Xaa-Pro bonds i.e. those bonds that are additionally retarded in their rotation by phosphorylation. A similar evolutionary need might have provoked the emergence of Par14-type parvulins. Neither Pin1- nor Par14-like parvulins have experienced an expansion during metazoan evolution as both fungi and mammals only contain one representative each. There is however an additional parvulin gene predicted in the genome of the green alga *Chlamydomonas reinhardtii*. The encoded protein contains a phosphobinding loop and an N-terminal forkhead domain instead of a WW moiety.^58^ Another survey on PPIase genes described a parvulin related protein of 44 kDa with a central parvulin domain in the fruit fly *Drosophila melanogaster*.^32^

### Par14 is localised to nucleus and cytosol and Par17 to mitochondria

There are only few hints for the function for Par14- type parvulins. The Par14 protein was initially detected within crude nuclear, cytosolic and mitochondrial fraction of human HEK 293 cells.^34^ Immunogold labelling revealed the protein to localise throughout the cell with certain enrichment in the nuclear matrix.^34^,^59^ At that time it was however unclear how this protein could reach the mitochondrial matrix. Independent follow up studies described Par14 within the cytosol and slightly enriched in the nucleus.^35^,^36^,^60^ Shuttling between these two cellular compartments is regulated by phosphorylation on Ser19 within the N-terminal flexible domain most likely by casein kinase 2.^35^ Although this part of Par14 is rich in basic residues, a classical nuclear localisation signal (NLS) could not be identified; N-terminal deletion studies though allowed narrowing the sequence down to Ser7 to Lys14 that functioned as NLS.^36^

A second parvulin protein species recently described might explain the amount of Par14 observed in mitochondria. By alternative transcription initiation an elongated messenger RNA is made at the parvulin locus on the X chromosome.^13^ This longer mRNA encodes an extended parvulin protein with additional N-terminal 25 residues, designated Par17 whose expression in human cells could be confirmed.^13^ The sequence between Met3 and Ala23 of Par17 forms an amphipatic α-helix that targets the protein to mitochondria where it is imported to the matrix in a membrane potential and time dependent manner.^14^ This protein might have resulted in the signals described above. Assuming that Par17 fulfils a similar function within the mitochondria as Par14 in the nucleus, this function would be limited to mitochondria of great ape species and man as only the genomes of these species do contain the Par17 coding sequence.^14^

### Par14 and Par17 as DNA-binding proteins

Within the nucleus, Par14 was reported to bind to double-stranded DNA.^36^ Based on certain similarity to the HMGB motifs in sequence-specific transcription factors like SRY and Lef-1 bent DNA oligonucleotides were tested for binding with Par14 resulting in AT-rich DNA octamers binding in the sub micro-molar range to Par14; the basic N-terminal part with sequence similarity to the chromatin-unfolding domain of HMGN proteins was indispensable for high affinity DNA binding.^36^ Such bent AT-rich segments of DNA are supposed to dictate nucleosome positioning 61 and play a role in transcription initiation suggesting involvement of Par14 in these processes. Par17 was equally well able to bind DNA at physiologic salt concentrations.^14^ Hence, it is reasonable to assume a function for Par17 associated with the mitochondrial nucleoid.

Besides DNA binding, Par14 was reported to bind to pre-ribosomal ribonucleoprotein particles.^60^ In GST pull down experiments followed by MS identification of associated biopolymers Par14 was reported to be part of the preribosomal ribonucleoprotein (pre-rRNP) complexes and as interacting with fibronectin, p160 (Myb-binding), p58 cyclindependant kinase (a G2/M-specific protein kinase) and α- and β-tubulin.^60^,^62^ Puzzling in this report was the finding that the basic domain alone was sufficient for most of these interactions. Therefore, the mode of interaction between Par14 and the just mentioned proteins might include relatively global ionic interactions. Taken together, the scarce available information on Par14 interactions in a cellular context suggest a function for this highly conserved protein in chromatin structure regulation, transcription and/or ribosome biogenesis.

## How to Target Par14-Type Parvulins?

Despite of a variety of studies regarding inhibitors of Pin1 that have been reviewed recently,^63^ there is only one study claiming the development of Par14 inhibitors.^39^ Within this study, a small directed compound library was screened for Pin1 inhibitors that was enriched in low-molecular-weight chemicals containing double-ring structures resembling the known, but unspecific parvulin inhibitor juglone.^64^,^65^ Candidate compounds were further derivatised yielding two Pin1 inhibitors with about 1.5 μM affinity that inhibited several Pin1 expressing cancer cell lines.^39^ The only argument for denoting theses two molecules also as Par14 inhibitors was molecular modelling using InsightII (Accelrys Inc) and Sybyl (Tripos Inc) softwares. These modelling studies indicated a supposedly fitting arrangement of side chains in Par14 also allowing binding of the reported Pin1 inhibitors. Both a direct interaction of these compounds with recombinant Par14 and the inhibition of its already weak PPIase activity still need to be demonstrated experimentally.

The attempt to develop competitive PPIase inhibitors against Par14 analogous to existing Pin1 modulators seems not very promising to us due to the hardly detectable PPIase activity described above and evidence that Par14 functions rather as a binding module than as a PPIase enzyme. In contrast, we here suggest the development of low-molecular-weight substances destabilising parvulin possibly by irreversibly reacting with the conserved cysteine 45 residue. Targeting the Par14 specific loop between Val103 and Asp107 that constitutes a putative protein-protein interaction surface together with the surrounding side chains might be another way of interfering with parvulin function. This could be achieved by specific small proteinous binders. Disturbing the cellular localisation of Par14 and Par17 proteins could be a third possibility to influence the function of these proteins.

## Figures and Tables

**Figure 3:**
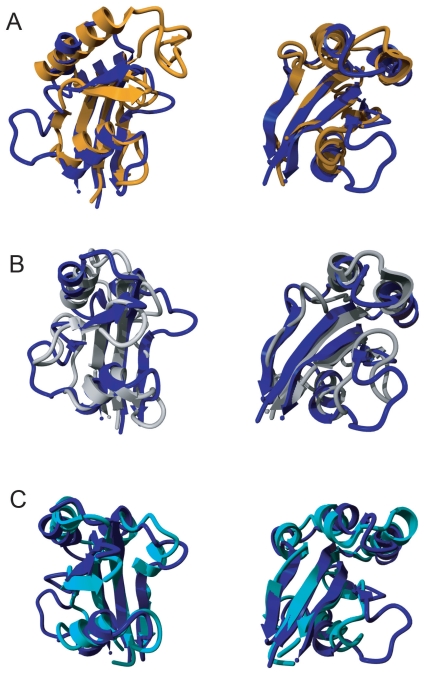
Structural comparison between different parvulin structures Overlay of the Par14 structure 1EQ3 in blue with A) Pin1 (1PIN) in orange; B) the Par14 structure 1FJD in gray and C) Par10 (1JNT) in cyan. The Par14 specific insertion is marked with an asterisk (*) in both views in A. All structural alignments were done with DALILite.^67^ Left column, front view; right column, back view.
